# Recognizing Coagulation Disorders in Sepsis in the Emergency Room: A Narrative Review

**DOI:** 10.3390/jcm15020488

**Published:** 2026-01-08

**Authors:** Toshiaki Iba, Tomoki Tanigawa, Hideo Wada, Kenta Kondo, Ricard Ferrer, Jerrold H. Levy

**Affiliations:** 1 Faculty of Medical Science, Juntendo University, Urayasu 279-0023, Japan; 2 Department of Emergency and Disaster Medicine, Graduate School of Medicine, Juntendo University, 2-1-1 Hongo, Bunkyo-ku, Tokyo 113-0033, Japan; tanigawa-tomoki@jbpo.or.jp (T.T.); k.kondo.be@juntendo.ac.jp (K.K.); 3 Medical Affairs Section, Research & Development Division, Japan Blood Products Organization, Tokyo 108-0023, Japan; 4 Department of General Medicine, Mie Prefectural General Medical Center, Yokkaichi 510-8561, Japan; wadahide@clin.medic.mie-u.ac.jp; 5 Intensive Care Department, Hospital Universitari Vall d’Hebron, Universitat Autònoma de Barcelona, 119-129 Barcelona, Spain; ricard.ferrer@vallhebron.cat; 6 Department of Anesthesiology, Critical Care, and Surgery, Duke University School of Medicine, Durham, NC 27710, USA; jerrold.levy@duke.edu

**Keywords:** disseminated intravascular coagulation, organ dysfunction, coagulopathy, intensive care unit, anticoagulants

## Abstract

Sepsis remains a leading cause of global mortality, and early management in the emergency department (ED) is a key determinant of clinical outcomes. Among the earliest physiological derangements in sepsis are abnormalities in coagulation, which represent not merely laboratory disturbances but fundamental reflections of dysregulated host response, endothelial injury, and evolving microvascular thrombosis. Sepsis-induced coagulopathy (SIC) and disseminated intravascular coagulation (DIC) form a dynamic continuum that frequently begins before shock is clinically apparent. Despite their prognostic value and pathophysiologic significance, these abnormalities are often underrecognized in the ED, where coagulation tests are still commonly interpreted through the narrow lens of bleeding risk rather than as markers of systemic thromboinflammation. This narrative review synthesizes current understanding of the mechanisms linking sepsis, endothelial dysfunction, and coagulation abnormalities; outlines the distinction between SIC and overt DIC; and highlights why early identification of coagulopathy in the ED is essential. We discuss practical bedside approaches, including recommended laboratory testing, pattern recognition, and application of validated scores such as the SIC and ISTH DIC criteria. System-level strategies, such as embedding coagulation testing into sepsis bundles, automating score calculation, and enhancing communication between the ED and ICU teams, are explored as avenues to improve early detection. Evidence suggests that ED recognition of SIC/DIC may refine risk stratification, guide triage decisions, and identify patients who may benefit from targeted anticoagulant strategies once stabilized. Ultimately, recognizing coagulation disorders in the ED reframes sepsis not solely as a hemodynamic crisis but as a complex, thromboinflammatory syndrome in which early intervention may alter trajectory and improve outcomes.

## 1. Introduction

Sepsis is defined as life-threatening organ dysfunction caused by a dysregulated host response to infection and remains one of the leading causes of death worldwide despite advances in critical care and antimicrobial therapy [1,2]. Emergency departments (EDs) represent the main entry point for patients with sepsis, and early decisions made in this chaotic environment strongly influence downstream intensive care unit (ICU) outcomes [3,4]. During this early phase, coagulation disorders serve as both a window into disease biology and a modifiable prognostic marker [5,6].

Sepsis-induced coagulopathy (SIC), which spans from mild abnormalities to overt disseminated intravascular coagulation (DIC), is common and strongly associated with organ failure and mortality [7,8]. Yet, in many EDs, coagulation testing is still primarily interpreted as a bleeding-risk assessment before invasive procedures, rather than as a dynamic marker of systemic thromboinflammation [9]. Recognizing SIC early, while patients are still in the ED, creates opportunities to refine risk stratification, tailor resuscitation, and consider anticoagulant strategies in selected patients [10,11,12,13]. This review highlights the pathophysiologic rationale, practical bedside recognition, and therapeutic implications of diagnosing coagulation disorders in sepsis at the point of care.

## 2. Literature Search Strategy

This narrative review was informed by a comprehensive and structured literature search aimed at identifying key evidence on coagulation disorders in sepsis, with a particular focus on early recognition in the EDs. Electronic searches were conducted in PubMed/MEDLINE, Embase, and the Cochrane Library from database inception to October 2025. The search strategy combined controlled vocabulary terms (MeSH and Emtree) and free-text keywords related to sepsis and coagulation abnormalities. Core search terms included “sepsis,” “septic shock,” “sepsis-induced coagulopathy,” “disseminated intravascular coagulation,” “coagulopathy,” “thromboinflammation,” “endothelial dysfunction,” and “emergency department”. These terms were used in various combinations with Boolean operators and adapted to the syntax of each database. Priority was given to systematic reviews, narrative reviews, randomized controlled trials, large observational studies, registry analyses, and international guideline or consensus statements, particularly those published by the International Society on Thrombosis and Haemostasis (ISTH), the Surviving Sepsis Campaign, and Japanese and multinational expert groups. Additional emphasis was placed on studies addressing pathophysiology, diagnostic criteria (ISTH DIC and SIC scores), prognostic implications, and therapeutic considerations, including anticoagulant strategies in sepsis-associated coagulopathy.

## 3. Pathophysiologic Bridge Between Sepsis and Coagulation

The intimate connection between inflammation and coagulation has been increasingly clarified over the past two decades [9]. In sepsis, pathogen- and damage-associated molecular patterns (PAMPs and DAMPs) activate innate immune cells, triggering a cytokine storm that upregulates tissue factor expression on monocytes and the damaged endothelium [7,8]. Thrombin generation is amplified, while physiological anticoagulant pathways, such as antithrombin, protein C, and tissue factor pathway inhibitor (TFPI), are suppressed, and plasminogen activator inhibitor-1 (PAI-1) levels rise, leading to impaired fibrinolysis [7,8,14].

**Figure 1:**
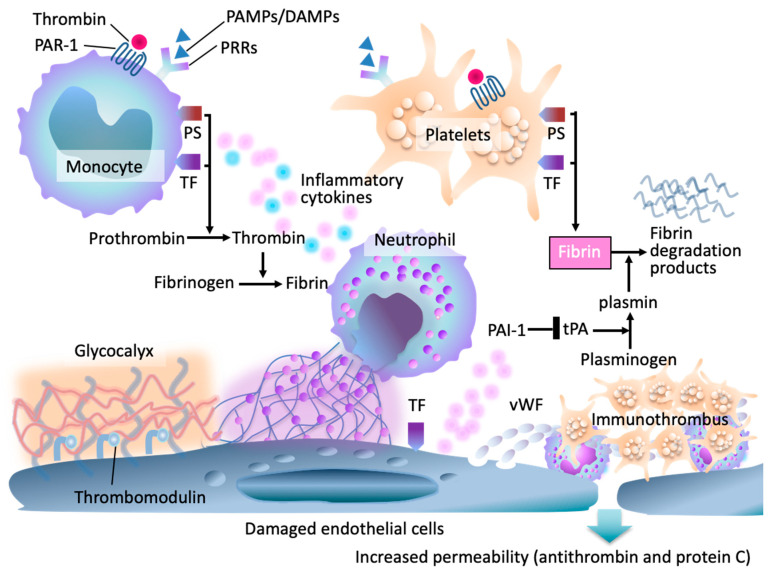
Pathophysiologic framework of sepsis-induced coagulopathy (SIC). In early sepsis, pathogen- and damage-associated molecular patterns (PAMPs/DAMPs) activate pattern-recognition receptors (PRRs) on monocytes, neutrophils, and endothelial cells, and upregulation of tissue factor (TF) and phosphatidylserine (PS). Endothelial injury results in degradation of the glycocalyx, increased permeability, and loss of endogenous anticoagulants, including antithrombin and the protein C system. Damaged endothelial cells release von Willebrand factor (vWF), leading to platelet adhesion and microvascular thrombosis. Thrombin generation amplifies through TF–factor VIIa complexes, further intensifying inflammation and coagulation. Impaired thrombomodulin-mediated anticoagulant activity and fibrinolytic suppression contribute to fibrin accumulation, fibrin degradation product release, and formation of immunothrombi.

The endothelium plays a central role in this process. Under physiological conditions, endothelial cells maintain an antithrombotic surface through the glycocalyx, thrombomodulin, and heparan sulfate [15]. In sepsis, inflammatory mediators and oxidative stress damage the glycocalyx, induce endothelial apoptosis, and convert the vascular surface into a pro-adhesive, procoagulant interface [16,17]. Microvascular thrombi form within multiple organs, resulting in the syndrome of thromboinflammation, in which coagulation is considered an effector arm of innate immunity, albeit at the expense of tissue perfusion [14,17,18] (Figure 1).

This continuum, from thromboinflammation to maladaptive, disseminated microvascular thrombosis, explains why even modest changes in platelet count, prothrombin time (PT)/international normalized ratio (INR), fibrinogen, and D-dimer can herald impending organ failure. From the ED perspective, these routine tests provide a readily available “early warning signal” of a systemic coagulopathy that is still potentially amenable to intervention [7,13,14,15,16,17].

## 4. From Sepsis-Induced Coagulopathy to Overt DIC

Historically, DIC was conceptualized as a binary entity, but contemporary frameworks emphasize a dynamic spectrum [8,9,10,11,14,19,20]. The ISTH DIC score, introduced in 2001 and updated in 2025, formalized widely used criteria that incorporate platelet count, PT prolongation, fibrin-related markers (fibrin/fibrinogen degradation products and D-dimer), and fibrinogen level [10,19]. While this score has high specificity for overt DIC, it was never designed to capture the earlier, compensated stages that are most relevant for ED decision-making [18].

To address this gap, the SIC score was proposed as an earlier marker of sepsis-associated coagulopathy, combining platelet count, PT-INR, and the Sequential Organ Failure Assessment (SOFA) score [12]. In large retrospective analyses, the SIC score identified patients at increased risk of progression to overt DIC and death, even when classical DIC criteria were not yet fulfilled [12,14]. The SIC concept, later incorporated into the ISTH Scientific and Standardization Committee communication, reinforces the idea that coagulopathy is not a late complication but an early hallmark of severe sepsis [14,18].

National and international guidelines further support the clinical relevance of DIC and SIC. Japanese guidelines and multinational reviews underscore that sepsis-associated DIC is common, carries a high mortality, and should be considered a therapeutic target rather than merely a laboratory curiosity [8,11,13,17]. From the ED vantage point, this means that the first abnormal coagulation results are not simply background noise but part of the core phenotype of high-risk sepsis.

## 5. Why Early Recognition in the Emergency Room Matters

Multiple observational studies have shown that the presence of coagulopathy in sepsis is associated with higher severity of illness and worse outcomes [7,8,13,14,17,18,21]. Patients meeting SIC or DIC criteria have significantly higher mortality than those without coagulopathy, even after adjustment for conventional severity scores [12,13,14,17,18]. In an era when EDs are expected to implement time-sensitive sepsis bundles, coagulopathy offers several advantages.

### 5.1. Prognostic Stratification at First Contact

Platelet count, PT-INR, and fibrin-related markers such as D-dimer are typically available within the first hour of ED presentation and provide important early prognostic information [22,23]. Observational studies consistently show that even modest abnormalities in these parameters are associated with greater illness severity, organ dysfunction, and mortality in sepsis [7,8,12,13,14]. Thrombocytopenia and PT prolongation reflect early activation of coagulation and endothelial injury, while elevated D-dimer indicates increased fibrin formation and turnover. Crucially, these changes often precede overt shock, allowing early identification of high-risk patients. In the ED, recognition of coagulation abnormalities can refine triage, support early ICU admission, and prompt closer monitoring, complementing conventional severity scores and enhancing early risk stratification [3]. However, further discussion of turnaround times and assay limitations is necessary.

Thromboelastography (TEG) and rotational thromboelastometry (ROTEM) are expected to offer the advantages of rapid turnaround times, bedside availability, and the ability to provide a global, real-time assessment of clot formation, strength, and fibrinolysis while acknowledging current limitations, including availability, cost, and lack of sepsis-specific thresholds [24].

### 5.2. Refinement of Sepsis Definitions and Organ Dysfunction

Sepsis-3 defines sepsis as life-threatening organ dysfunction quantified by an increase in the SOFA score, in which coagulation, reflected by platelet count, is a core component rather than an ancillary variable [1]. This framework highlights that coagulation abnormalities are intrinsic to organ dysfunction in sepsis, rather than being merely laboratory bystanders. Incorporating explicit awareness of SIC and DIC into ED workflows, therefore, aligns directly with contemporary sepsis definitions. Subtle but progressive thrombocytopenia or mild prolongation of prothrombin time may represent early microvascular and endothelial injury and should not be dismissed as nonspecific findings [7,8,25]. When interpreted dynamically and in a clinical context, these changes can signal evolving organ dysfunction before overt shock, reinforcing their value in early risk stratification and decision-making in the ED [1,12,13,14].

### 5.3. Therapeutic Window for Anticoagulant Strategies

The debate over anticoagulant therapy in sepsis-associated DIC remains unresolved; however, there is a broad consensus among experts that timing and appropriate patient selection are critical determinants of efficacy [13,17,18] (Table 1). Although the trials were largely ICU-based and conducted at advanced stages of sepsis, their findings remain highly relevant to ED practice. The predominantly neutral results likely reflect delayed enrollment, advanced organ dysfunction, and a lack of early biological stratification, rather than the failure of coagulation-targeted therapies. From an ED perspective, these data highlight the importance of early identification of sepsis-induced coagulopathy, when coagulation and endothelial abnormalities may still be biologically modifiable and precision-guided intervention more plausible.

Accumulating evidence suggests that anticoagulant interventions are most likely to confer benefit when initiated during the compensated or early decompensated phases, when microvascular thrombosis predominates, and bleeding risk remains acceptable [35,36,37,38]. Once patients progress to profound thrombocytopenia, overt bleeding, or advanced multiorgan failure, the therapeutic window rapidly narrows [39]. Early identification of SIC or evolving DIC in the ED, therefore, establishes the earliest clinically actionable window for risk stratification and downstream consideration of targeted anticoagulant strategies [12,13,14,17,18].

### 5.4. Integration with Infection Source Control and Resuscitation

As shown, coagulopathy in sepsis reflects not only the intensity of the systemic inflammatory response but also the severity, burden, and chronicity of the underlying infection [14,40]. Persistent or progressively abnormal coagulation variables, such as worsening thrombocytopenia, rising PT/INR, or markedly elevated D-dimer, may indicate ongoing pathogen load, delayed source control, or tissue ischemia driving sustained thromboinflammatory activation [7,8,14]. In the ED, such patterns should prompt clinicians to reconsider the adequacy of initial diagnostic assumptions and pursue a more aggressive search for occult infection sources, including intra-abdominal catastrophe, ischemic bowel, or deep-seated abscesses [25]. Recognition of evolving coagulopathy may also lower the threshold for early cross-sectional imaging, surgical consultation, or escalation of care, supporting timely source control and improved outcomes [4,8,11].

## 6. Practical Bedside Recognition: A Simple ED Framework

**Figure 2:**
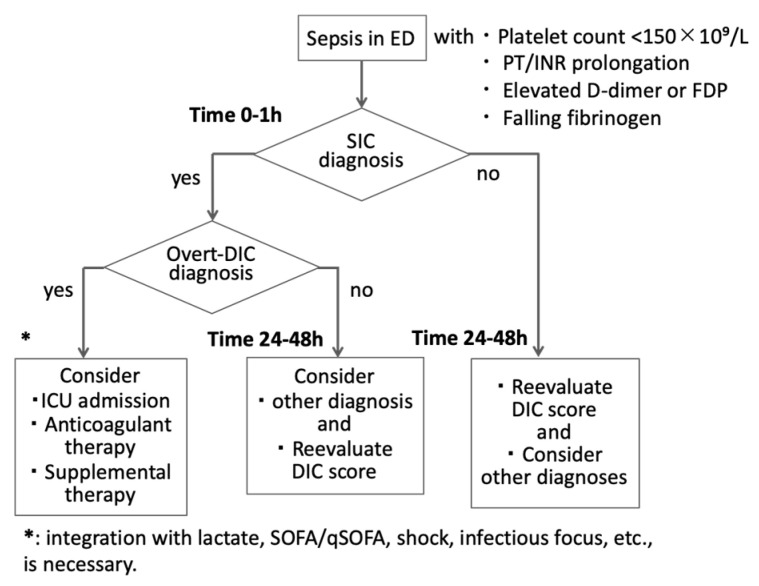
Emergency department diagnostic flow for coagulopathy in suspected sepsis. In septic patients, an initial coagulation panel (platelet count, prothrombin time [PT]/international normalized ratio [INR]) should be obtained within one hour after emergency room presentation (Time 0–1 h). Once patients are diagnosed with sepsis-induced coagulopathy (SIC), further testing, such as fibrin/fibrinogen degradation products (FDP), D-dimer, and fibrinogen, is necessary to confirm the diagnosis of disseminated intravascular coagulation (DIC). Because SIC and DIC may evolve after initial sampling, repeat testing at 24–48 h is essential in patients with persistent infection or organ dysfunction. Coagulation results inform risk stratification and early ICU consultation. Therapeutic considerations (e.g., anticoagulants) should be taken into account, and a bleeding risk assessment is warranted. SOFA: sequential organ failure score, qSOFA: quick SOFA.

In busy ED practice, a pragmatic, stepwise approach is needed (Figure 2). A feasible framework incorporates the following:

### 6.1. Routine Screening

Early ED coagulation panel within 1 h, including platelet count, PT/INR, for the diagnosis of SIC and used for risk stratification and ICU triage. The tests should be repeated at 24–48 h for evolving cases. For patients who fulfilled SIC, the coagulation panel should be ordered early in the course of evaluation to confirm DIC. This includes a complete blood count with platelet count, PT-INR, fibrinogen concentration, and fibrin-related markers such as D-dimer or FDP [11,22,23,41]. Together, these readily available tests enable the early identification of sepsis-associated coagulopathy and the progression to DIC. Although full coagulation testing with repeat sampling in high-risk infection sources is ideal, it is understandable that recommendations change depending on the country’s situation. It is also recommended that SIC (and, when data permit, DIC) scoring be calculated as soon as core labs return, ideally within the first ED evaluation window, and reassessed serially when the illness is evolving, or initial results are borderline. Importantly, such testing is already endorsed in multiple national and international DIC guidelines and can typically be incorporated into routine sepsis blood panels without incurring high additional costs, delays, or logistical burdens in the ED setting [6,7,8].

### 6.2. Rapid Pattern Recognition

The following patterns should trigger concern for early sepsis-associated coagulopathy: platelet count <150 × 10^9^/L or a rapid downward trend on serial sampling, PT/INR prolongation beyond local reference ranges, markedly elevated D-dimer or FDP, and a decline in fibrinogen, particularly in the context of rising D-dimer [42].

Even if individual abnormalities are modest, their combination and trajectory are important. An ED patient with mild thrombocytopenia, slightly prolonged PT, and very high D-dimer likely sits on the early SIC to DIC continuum, even if overt DIC scores are not yet positive [8,12,13,14].

### 6.3. Use of Simple Scores

Where feasible, ED teams can apply SIC and ISTH DIC scores (Table 2).

- SIC score: based on platelet count, PT-INR, and SOFA; a score ≥4 has been associated with increased mortality and progression to overt DIC [12,14].
- ISTH DIC score: more specific for overt DIC but still helpful as a baseline, especially if repeated during the first 24–48 h [10,11].

Even if formal scoring is deferred to the ICU, the ED can at least flag patients with likely SIC by noting thrombocytopenia plus PT/INR prolongation in the context of organ dysfunction [12,13,14,18].

### 6.4. Communication and Documentation

Recognition of coagulopathy alone is insufficient unless it is clearly communicated across care transitions. ED documentation should explicitly note when a patient presents with sepsis complicated by coagulopathy (suspected SIC or DIC), rather than listing isolated laboratory abnormalities. Clear labeling ensures that intensive care unit teams maintain serial monitoring of coagulation parameters, reassess trajectories over time, and incorporate coagulopathy into ongoing risk stratification. Such explicit communication also facilitates timely, multidisciplinary discussions regarding bleeding risk and the potential role of anticoagulants or supportive therapies as the patient’s clinical course evolves [8,13,17,18].

## 7. Therapeutic Implications: What Can the ED Influence?

The primary treatment of sepsis-associated coagulation disorders remains optimal sepsis care, which includes timely antibiotics, adequate fluid resuscitation, vasopressor support, and source control [1,4]. However, accumulating evidence has led to renewed interest in targeted anticoagulant therapies in selected patients with SIC or DIC [43].

### 7.1. Anticoagulant Strategies in Sepsis-Associated DIC

Clinical trials of antithrombin, recombinant thrombomodulin, and heparin in unselected sepsis populations have produced mixed or negative results, but subgroup analyses and registry data suggest potential benefit in patients with definite DIC [13,17,18]. Recent reviews highlight the following:

- Anticoagulant therapies may reduce mortality and improve DIC scores in carefully selected patients with sepsis-induced DIC, particularly when therapy is initiated early, and bleeding risk is low [13,17,21].
- The risk–benefit balance shifts unfavorably once patients develop severe thrombocytopenia, active bleeding, or advanced organ failure [13,17,21].

For the ED clinician, the key question is not which specific anticoagulant to start immediately but whether the patient should be considered a potential candidate for such therapy after initial stabilization. Early identification of SIC/DIC in the ED ensures that by the time ICU teams evaluate anticoagulant options, the patient will not have “missed” the therapeutic window [12,13,14,17,18,21].

### 7.2. Guideline Perspectives and Regional Differences

The global landscape of sepsis-associated coagulopathy reflects historically distinct clinical philosophies that are now beginning to converge. Differences between the Japanese and Western approaches have been particularly visible, shaped by variations in research traditions, healthcare infrastructure, and tolerance for therapeutic risk. In Japan, sepsis-associated coagulopathy, including SIC and overt DIC, has long been regarded not merely as a laboratory abnormality, but as a biologically actionable process. This perspective is supported by decades of nationwide observational studies and registry data, which have promoted the structured use of DIC screening and the selective application of antithrombin and recombinant thrombomodulin in carefully phenotyped, high-risk patients [8,11,13,17,21,43,44]. Consequently, SIC and DIC are commonly monitored, interpreted longitudinally, and incorporated into therapeutic decision-making rather than treated as passive markers of severity [45].

In contrast, Western guidelines have traditionally adopted a more cautious stance. Influenced by large randomized trials conducted in heterogeneous ICU populations and by concerns regarding bleeding risk, professional societies have emphasized early supportive care and infection control, while generally discouraging routine anticoagulant therapy outside of clinical trials or exceptional circumstances [4,10,13,14,46]. In many Western settings, coagulopathy has therefore been viewed primarily as a prognostic indicator rather than a direct therapeutic target. ED risk stratification has accordingly focused on rapid clinical tools such as qSOFA and early warning scores, which prioritize organ dysfunction and mortality risk but do not specifically capture evolving thromboinflammatory biology.

Despite these differences, recent international reviews and consensus statements suggest growing alignment around several key principles [14,17,18,47,48,49]. There is increasing recognition that early identification of SIC and DIC is important not only for prognostication, but because it signals a transition toward microvascular injury and endothelial dysfunction—processes that precede overt shock and may be biologically modifiable [12,13,18]. Similarly, experts increasingly emphasize that coagulation parameters should be assessed dynamically, with serial measurements providing greater insight than isolated values obtained solely to evaluate bleeding risk [50]. While uncertainty remains regarding optimal anticoagulant strategies, a cautious consensus is emerging that selected patients with early SIC/DIC and low bleeding risk may benefit from targeted interventions, provided that timing, dosing, and patient selection are refined in future trials [44].

These evolving concepts have particular relevance for the ED, which represents the first point of contact for many patients at risk of developing clinically significant coagulopathy. Rather than functioning as an isolated diagnostic environment, the ED serves as a critical interface between early risk stratification and downstream ICU or hematology-led management. Harmonized institutional pathways—integrating rapid clinical scores (e.g., qSOFA), early coagulation assessment, and structured communication using SIC or DIC frameworks where feasible—offer a pragmatic means of bridging regional practice differences. In this way, sepsis-associated coagulopathy is increasingly recognized not as a region-specific construct, but as a universal dimension of sepsis biology that demands early, coordinated, and context-sensitive recognition across healthcare systems.

## 8. Systems-Level and Research Implications

From a systems perspective, embedding coagulation assessment into the early management of sepsis in the ED represents a natural evolution of modern sepsis care. As the understanding of sepsis has shifted from a purely hemodynamic disorder to a complex syndrome marked by dysregulated inflammation, endothelial injury, and microvascular thrombosis, it has become increasingly clear that abnormalities in platelet count, coagulation times, and fibrin-related markers are not peripheral laboratory curiosities but early reflections of disease biology [7,8,14,17,18,51,52,53].

Introducing standardized coagulation testing into ED sepsis bundles, much like the now-routine use of lactate, could fundamentally change how clinicians stratify risk and anticipate deterioration. Automated calculation of SIC or DIC scores through electronic health record systems would enable abnormalities to be promptly flagged, even in busy clinical environments. Such alerts could trigger early communication with critical care or hematology teams, mirroring successful early-warning approaches used for sepsis and organ failure in many health systems [3,14,54,55].

Coagulopathy profiles could also be incorporated into institutional quality metrics. Linking SIC/DIC recognition with outcomes, such as ICU admission rates, the need for vasopressor support, or mortality, would create a feedback system that supports continuous quality improvement and encourages clinicians to consider coagulation as a core physiological axis, rather than an afterthought. Importantly, this approach aligns with emerging data showing that endothelial dysfunction and microvascular thrombosis develop early in sepsis and strongly predict clinical trajectory [16,17].

Although much of the current evidence is derived from ICU-based studies, these datasets offer a framework for conducting similar analyses starting at ER arrival. Large registries have demonstrated that coagulopathy is independently associated with mortality across diverse sepsis populations; however, the prognostic value may be even greater when measured in the first hours of illness [7,8,17,18]. This suggests several high-yield research priorities. First, can early ED identification of SIC or DIC improve triage accuracy—helping clinicians decide earlier who needs closer monitoring or expedited ICU transfer? Second, does systematic SIC screening enable earlier initiation of anticoagulant strategies in selected patients, potentially improving outcomes as suggested by emerging post-hoc analyses and observational data [13,17,21,30]. Third, we still lack robust analyses on how different infection sources, host immune phenotypes, age groups, or chronic comorbidities influence the prognostic value of ED-identified coagulopathy—particularly relevant given the heterogeneous nature of sepsis endotypes [21,25,26,56,57,58].

Answering these questions will require coordinated research across emergency medicine, critical care, and hematology. Multicenter ED-based sepsis registries incorporating SIC and DIC variables, endothelial biomarkers, and microcirculatory assessments may help clarify which patients benefit most from targeted therapies [52]. As precision medicine approaches continue to evolve, the ED has the potential to become the earliest point at which clinicians can identify thromboinflammatory phenotypes and shape downstream therapy—long before microvascular injury becomes irreversible.

## 9. Conclusions

Coagulation abnormalities in sepsis should not be viewed as obscure laboratory findings that surface only once a patient reaches the ICU. In reality, they are woven into the very fabric of sepsis pathophysiology and often begin to unfold long before shock becomes clinically obvious. In many patients, the first signs of microvascular thrombosis, platelet consumption, or fibrinolytic shutdown are already present when they arrive at the ED. This makes the ED a uniquely important setting, arguably the most important, for detecting sepsis-associated coagulopathy at a moment when it is still evolving and potentially reversible.

When emergency clinicians incorporate coagulation testing as a routine part of sepsis evaluation, the early landscape of the disease becomes clearer. Even simple tools such as the SIC score or the ISTH DIC score can transform scattered laboratory values into a coherent physiological signal. More importantly, when these findings are recognized and communicated promptly to admitting teams, they influence decisions about triage, monitoring intensity, and the possible role of anticoagulant therapies in selected patients.

Through this lens, coagulopathy is no longer a downstream complication but a window into the biological momentum of sepsis—from inflammatory activation to microvascular dysfunction. Identifying these changes early helps clinicians sharpen risk stratification, anticipate deterioration, and align ED practice with the rapidly evolving international evidence base.

Ultimately, recognizing coagulation disorders in the emergency room is an exercise in seeing sepsis for what it truly is: a complex, dynamic interaction between inflammation, coagulation, and organ perfusion. By appreciating these mechanisms from the very first encounter, clinicians take an important step toward delivering more precise, mechanism-guided care—care that has the potential to alter the trajectory of one of medicine’s most challenging syndromes.

## Figures and Tables

**Table 1:** The major RCTs of systemic anticoagulant therapy in sepsis.

Anticoagulant/Trial	Target Population	DIC/SIC Inclusion Criteria	*n*	Primary Endpoint	Main Findings	Ref.
**Antithrombin—KyberSept**	Adults with severe sepsis or septic shock within 6 h of onset	No formal DIC/SIC requirement; sepsis defined by infection + ≥1 organ dysfunction	2314	28-day all-cause mortality	High-dose AT (30,000 IU over 4 days) did not reduce 28-day mortality; more bleeding, especially with concomitant heparin.	[26]
**Antithrombin—JAAM-DIC AT trial**	Sepsis-induced DIC in Japanese ICUs	JAAM DIC score ≥ 4 and AT activity 50–80%	60	Change in DIC score and 28-day mortality (exploratory)	Moderate-dose AT (30 IU/kg/day × 3 days) improved DIC scores/DIC resolution without excess bleeding, but no mortality benefit (underpowered).	[27]
**Activated protein C** **—PROWESS**	Severe sepsis with ≥1 acute organ failure	No DIC/SIC requirement; coagulopathy common but not required	1690	28-day all-cause mortality	Drotrecogin alfa (24 µg/kg/h × 96 h) reduced 28-day mortality (30.8% → 24.7%), but at the cost of more serious bleeding	[28]
**Activated protein C** **—ADDRESS**	Severe sepsis, lower risk of death(APACHE II < 25 or single-organ failure)	No DIC/SIC requirement	≈2600	28-day mortality	In low-risk patients, drotrecogin alfa did not reduce mortality; possible harm in the very-low-risk subgroup.	[29]
**Activated protein C** **—PROWESS-SHOCK**	Septic shock on vasopressors ≥ 4 h	No DIC/SIC requirement	1696	28-day and 90-day mortality	No mortality benefit vs. placebo; similar bleeding.	[30]
**TFPI (tifacogin)** **—OPTIMIST**	Severe sepsis with at least one organ failure	No DIC/SIC requirement; some post-hoc DIC analyses	1754	28-day mortality	Tifacogin did not reduce mortality overall; increased serious bleeding.	[31]
**Thrombomodulin (ART-123)** **—Phase 2b**	Sepsis with suspected DIC	Modified ISTH DIC (prolonged PT, low platelets, elevated FDP/D-dimer)	750	28-day mortality	ART-123 0.06 mg/kg/day × 6 days: no statistically significant mortality reduction; post-hoc signal in more severely coagulopathic subgroup.	[32]
**Thrombomodulin** **—SCARLET**	Sepsis-associated coagulopathy with CV or respiratory failure	INR > 1.40 and platelets 30–150 × 10^9^/L, plus sepsis with organ failure (SIC-like phenotype)	800	28-day all-cause mortality	Recombinant TM did not significantly reduce 28-day mortality (26.8% vs. 29.4%); numerical trend but primary endpoint negative.	[33]
**UFH** **–HETRASE**	Severe sepsis in a general ICU; not DIC-enriched	No formal DIC/SIC criteria	155	28-day mortality and safety	Low-dose IV heparin (12 IU/kg/h × 7 days) showed a non-significant trend toward lower mortality, more ventilator-free days; no clear increase in major bleeding; underpowered.	[34]

DIC: disseminated intravascular coagulation, SIC: sepsis-induced coagulopathy, AT: antithrombin, APACHE II: Acute Physiologic Assessment and Chronic Health Evaluation II, TFPI: tissue factor pathway inhibitor, ICU: intensive care unit, CV: cardiovascular.

**Table 2:** ISTH overt DIC (2001, 2025) and SIC scoring systems.

Item		Overt DIC 2021	Overt DIC 2025	SIC
	Score	Range	Range	Range
**Platelet count** **(× 10^9^/L)**	2	<50	<50	<100
	1	≧50, <100	≧50, <100	≧100, <150
**D-dimer**	3	Strong increase (or FDP)	> × 7 upper normal limit	−
	2	Moderate increase (or FDP)	> × 3 upper normal limit	−
	1	−	−	−
**Prothrombin time prolongation/INR (PT** **-INR)**	2	≧6 s	≧6 s	>1.4.
	1	≧3 s, <6 s	≧3 s, <6 s	>1.2, ≦1.4 (PT-INR)
**Fibrinogen** **(mg/dL)**	1	<100	<100	−
**SOFA score**	2	−	−	≧2
	1	−	−	1
**Total score for DIC**		≧5	≧5	≧4

ISTH, International Society on Thrombosis and Haemostasis; DIC, disseminated intravascular coagulation; SIC, sepsis-induced coagulopathy; SOFA, sequential organ failure assessment; INR, international normalized ratio. The total SOFA score is the sum of 4 items (respiratory SOFA, cardiovascular SOFA, hepatic SOFA, and renal SOFA). The total score of platelet count and PT-INR must exceed 2 for the diagnosis of SIC.

## Data Availability

No new data were created or analyzed in this study.
